# Experimenting with visual content: online focus group on citizens’ perception and trust in science communication

**DOI:** 10.12688/openreseurope.14621.2

**Published:** 2023-03-30

**Authors:** Chiara Piccolo, Giuseppe Pellegrini, Marina Tulin, Gábor Szüdi, Pamela Bartar

**Affiliations:** 1 Observa - Science in Society, Viale Fusinieri, 65, Vicenza, 36100, Italy; 2 University of Amsterdam, Nieuwe Achtergracht, 166, Amsterdam, 1018, The Netherlands; 3 ZSI - Zentrum für Soziale Innovation, Linke Wienzeile 246, Wien, 1150, Austria

**Keywords:** science communication, trust, visual communication, workshop, sound and image

## Abstract

**Background: **This paper presents part of a wider research project called TRESCA[1] which aims to develop trust in science through the innovation of communication practices.

Connected with the topic of trust in sciences, in terms of the credibility and reliability of scientific information, a part of the project was dedicated to the assessment of the explanatory power of two main elements of communication: audio and video. Particular attention was given to how these two elements relate to the perception of citizens, mediating with the latent imaginaries, emotional charges and value judgements that are the basis of the framing of relevant news, and thus with the ability of people to distinguish between correct and false communication.

**Methods: **To investigate these aspects, an *ad hoc *workshop was designed and implemented in three European countries with the aim of understanding how people interpret the content of a scientific communication video with particular attention to the role of images and audio. Some probe questions were carefully selected to explore content and latent imaginaries, emotional and critical aspects also related with the trust of the communication.

**Results: **By involving citizens in discussions and innovation efforts, many suggestions and recommendations have been collected. In today’s everyday life, where the visual is very widespread, thanks to the ever-growing presence of social media, the power of images can exceed that of audio.

**Conclusions: **Since watching a video without sound can mislead the real content of the message, especially when the source or the speaker aren’t recognisable. This can have many repercussions on people’s ability to evaluate the truthfulness of a news and, consequently, on the choice to grant trust - or distrust.

## Plain language summary

TRESCA - *Trustworthy, Reliable and Engaging Scientific Communication Approaches* - is a research project on the trust that people place in science and its main purpose is to understand how to increase this trust by improving communication practices. Indeed, communication is made up of many different factors that can influence an impact of trust or mistrust, especially in these times of visual communication and the widespread use of digital media.

A part of TRESCA was dedicated to the evaluation of two specific components of communication: audio and video. Attention was focused on how these two components relate to the perception of a message and therefore to people's ability to distinguish between correct and false communication. We know from previous research that specific elements such as style, format, the activation of a collective imagination - the way people think about something - and emotional reaction can influence the perception of a message.

To investigate these aspects, a workshop was designed and then carried out in three different European countries. Within the workshop, some probe questions were selected to explore style and format, content, collective imagination and emotional reactions related to two videos on the Covid-19 issue that the participants saw first without audio and then with audio. This double watching made it possible to highlight how the audio and video component relate to the other elements mentioned.

By involving citizens in the discussions, many suggestions and recommendations were collected. In general, the power of images can exceed that of audio. However, watching a video without sound can mislead the actual content of the message, especially when the source or speaker is not recognizable. This can have many repercussions on people's ability to evaluate the truthfulness of a news and, consequently, on the choice to grant trust - or distrust.

## Introduction

The global pandemic amplified the relevance of science communication in society and prompted researchers to focus even more on the relationship between science and society ( Metcalfe *et al.*, 2020). Some of the questions that have a central role in the current debate on scientific communication are: What kind of science is being communicated? What image of science do we have in mind when we turn our interest to science communication? ( Besley & Nisbet, 2013).

On the other hand, society is increasingly visual and the visual has become a big part of our daily life, especially through the internet and social media ( Adami & Jewitt, 2016; Fahmy *et al.*, 2014). In fact, the ever-growing presence of social media and other digital tools that populate the web has increased the production and reception of visual stimuli capable of changing the way we understand social relations and life in organisations ( Davison *et al.*, 2015). Through the media, in particular the tools of the internet and social media, the public builds an image of objects, people, social relationships and norms. Studies conducted by Scheufele and Iyengar permit to verify to what extent the media can influence public opinion ( Scheufele & Iyengar, 2017). For example, new digital technologies (e.g., morphing techniques) can be applied to measure precisely which manipulation leads to specific effects. In this way, some algorithms related to the use of big data and in particular to Facebook, facilitate the study of which visual messages allow the public to act autonomously while maintaining a critical look.

In this new media ecosystem, the strength of images has become so relevant that it is attracting the attention of more scholars and a vast amount of literature on the topic has already been produced ( Hermann *et al.*, 2021; Kümpel, 2019; León & Bourke, 2018; Meier *et al.*, 2020; Trillò *et al.*, 2021).

Among the most relevant theories on the influence of visual communication is the *Framing Theory* ( Nilsson, 2015), a theory that highlights the ability of images to convey messages in an implicit and symbolic way. According to prominent theorists of this perspective, framing is a potentially useful paradigm for examining the strategic creation of messages in public relations and the consequent reactions of the public ( Fahmy, 2004; Kress & Van Leeuwen, 2001; Parry, 2010).

Visual communication therefore proposes roles, behaviours, norms and values that profoundly affect the imagination of the public. Studies on the subject of credibility and trust in sources show that the reputation of the source is an important credibility heuristic ( Metzger *et al.*, 2010), and that credibility lies foremost in the trustworthiness and expertise of the source itself ( Tseng & Fogg, 1999). Users tend to transfer the reputation of the source (companies as well as news organisations) to the content itself ( Metzger *et al.*, 2010). The analysis of different sources of information shows that the public has a certain level of scepticism with regard to the reliability of the content produced on Facebook and Twitter ( Fletcher & Nielsen, 2018). Data from the Science, Technology and Society National Observatory of Observa, for example, confirm that in Italy only 4% of the population considers the scientific news presented on social media to be very credible ( Bucchi & Saracino, 2022). In general, it can be said that information from institutional sources is more credible while information from individuals is viewed with greater suspicion.

The TRESCA project’s main objective is to study how more effective communication approaches can be developed in a time of great media changes by analysing the mechanisms that facilitate the development of trust - or mistrust - in science. As part of the project, three workshops were carried out with citizens in three different countries: Italy, the Netherlands and Austria. The aim of these workshops was to understand how people interpret the content of a science communication video, paying particular attention to the role of images and audio. Using two videos relating to Covid-19 as stimulus and listening to citizens’ reactions and judgements, the TRESCA project team was able to explore what a good visual science communication format focused on the role of images and audio should look like. The proposed activity was designed as a quasi-experiment. Unlike a real experiment, a quasi-experiment is characterised by a series of features, as indicated by Campbell & Stanley (1963): manipulation of the variables examined, the absence of a control group, the non-randomness of the selection of the subjects involved and the formulation of hypotheses that indicate a relational link between associative variables (covariation).

In other words, this study does not claim to be exhaustive given its experimental character. Rather, it intends to provide some useful indications to proceed with the study of trust mechanisms that citizens build through scientific communication.

## Methods

### Study design

In order to analyse the mechanisms of the communication that may facilitate the development of trust in science, a specific experimental workshop was implemented. This tool was designed by Vincenzo Pavone, a member of the *Consejo Superior de Investigationes Cientificas* (CSIC) ^2^ project team and tested through a pilot event.

In particular, the workshop aimed to understand how people interpret the content of science communication, paying attention to the visual and audio content. For this purpose, two videos on the topic of Covid-19 were chosen and the workshop was structured in three main phases. In the first phase participants watched the videos without sound and had the opportunity to share their views and impressions. Then, they watched the same videos with sound, discovering their actual content and had again the opportunity to share their impressions. Afterwards, participants were invited to use a fact-checking service to find information on the reliability of the information provided in the videos, after which they had another chance to share their ideas. A final discussion concluded the workshop by collecting suggestions and recommendations.

The choice of removing the sound in the first phase aims at exploring the impact of visual communication on communication perception, particularly emotional responses as well as perceptions of the topic and images. The comparison between participants’ reactions to the videos without and with sound helped the TRESCA project team distinguish between the impact of images and words on participants’ perceptions of the science communication videos.

### Tools and procedure

Two videos were carefully selected from a list of thirty videos and were tested during a pilot activity. These two videos were chosen because, more than the others, they dealt with the same issue in a different way and with different communication styles, thus representing two exemplary extremes of opposite formats. As mentioned in Table 1, the first video ^3^ presents a scientific communicator arguing against the relationship between Covid-19 and 5G technology; the image sequence and the formal sounding are very fast and loud. The second video ^4^, on the other hand, shows an online group interview where people talk about their direct experience of getting infected with Covid-19; this second video is slower and calmer in both audio and visual content.

**Table 1.** Details of the videos used in the workshops.

Video	Description
Video number 1: “No, 5G Is Not Causing Corona virus (or Anything Else)” https://www.youtube.com/watch?v=KassIV7qLGk	Documentary video: viral online posts claiming 5G is causing Corona virus are absolutely wrong. Conspiracy theorists are taking them seriously, however, and some are turning violent. Here’s why their arguments are nonsense. ● Images and other videos included ● Generally clear semantic ● Understandable ● For now, just auto generated English subtitles ● No cuts needed ● Misinformation *Time: 3 mins*
Video number 2: “Do not ignore this: COVID-19 survivors speak out during DeLand round table” https://www.youtube.com/watch?v=FlH5DKNsuy0	Talk show video “Do not ignore this”: COVID-19 survivors speak out during DeLand round table ● Images ● Understandable ● Different perspective and experiences ● No cuts needed ● For now, just auto generated English subtitles *Time: 2 mins*

As mentioned, after watching the videos, both in the first (without audio) and in the second phase (with audio), the participants had the opportunity to share their opinions and impressions. The discussion in these stages was facilitated by a moderator ^5^ who made use of probe questions. In particular, the questions in the first phase were: *What do you think was the content of these videos? How would you describe the emotions that each of them made you feel? What are the images in each video that triggered your curiosity or that you remember better? Do you think the videos are about science communication or not? Who do you think the people in the videos are?*

The questions in the second phase were: *Now, you know what the videos were about, how do you feel? How has the sound changed your perceptions and interpretation of the content? What do you know about the topic discussed in the video? Do you think the way the information was presented was effective? Who do you think the narrator is? Do you think the narrator was trustworthy?* The interview guide can be found under *Extended data*. ^6^

These questions helped the team to investigate the most relevant issues surrounding scientific communication and communication in general in line with the objectives of the TRESCA project.

Due to the pandemic situation, the workshop took place online through the Zoom video conference service and audio and visual recording was used to collect data in addition to any field notes collected by the moderators. The final structure of the workshop is schematically shown in Table 2 with details of the duration of each session. Thus, participants gathered in the main virtual room for a brief introduction to the event before they could join their respective groups in the smaller breakout rooms. Once in the breakout rooms, each group could begin with the video watching and the discussion sessions as described above. Finally, all participants came back to the plenary virtual room and discussed what emerged during the group sessions, making suggestions and offering recommendations on how to improve visual scientific communication. Before the participants left the meeting, they were asked to answer the evaluation questionnaire of the organisational and managerial aspects of the workshop. A copy of the questionnaire can be found under *Extended data*. ^7^

**Table 2.** Workshop Agenda.

Timing	Activity
10 minutes	Introduction and presentation
40 minutes	First phase: Video without sound and group discussion (Zoom breakout rooms)
15 minutes	Break
40 minutes	Second phase: Video with sound and group discussion (Zoom breakout rooms)
5 minutes	Third phase: Website news checking
30 minutes	Fourth phase: Recommendation
10 minutes	Final questionnaire and conclusion

The method made it possible to detect the effect of audio and visual elements on people’s perceptions through their active involvement. People discussed the role of emotions, the credibility of the information provided, and the role of the different people appearing in the videos. Listening to the participants’ reactions on the videos without and with sound, it was possible to better recognize how people build their understanding of science, their previous beliefs, their standpoint, and the elements that influence their trust in scientific communication.

### Participants

The workshop was implemented in three different European countries: Italy, the Netherlands and Austria. Each event took place on a different day, these were chosen by each partner based on its national cultural habits and ideal time-line to maximise citizens’ engagement and participation.

The Italian Workshop was organised by Observa ^8^ and involved a total of 34 participants, divided into four groups. The Dutch Workshop was managed by EUR ^9^ and was comprised of a total of 13 participants, split into three groups. The Austrian Workshop was facilitated by ZSI ^10^ and was comprised of a total of 17 participants, divided into four groups.

The recruitment process in the three countries was conducted using two methods and different channels to approach potential participants. In all countries involved, the snowball technique and targeted emails were implemented to involve participants; in particular several channels were used to do these: newsletters, websites and social media (e.g. LinkedIn, Twitter and Facebook) connected to the institutional profiles of the three partners and to the consortium networks. In addition, for the Italian case, a reserve list of participants was prepared to replace those who could not participate; for the Dutch workshop, an advertisement was created that included all the information and registration URL to easily spread the details on different channels. Finally, press articles were published in Austria through the channel of the Austrian Press Agency (APA Science) and the ZSI website.

Participants were adult volunteers (18 years old or above) who were in the position to understand and consent to the proposed research ^11^. During the recruitment process, the local organizing partners took into account many socio-demographic variables ^12^. Specifically, the Italian participants were 21 women and 13 men; 14 aged between 18 and 34, 15 aged between 35 and 54, and 5 aged over 55; 3 of them have got a primary or lower secondary education, 13 a secondary school diploma and 18 a university degree. For the Netherlands there were 6 women and 7 men; 6 aged between 18 and 34, 1 aged between 35 and 54 and 6 aged over 55; 1 person has a secondary school diploma and the others (N=12) have a university degree. Regarding the Austrian participants, they were 12 women and 5 men; 7 aged between 18 and 39, 6 aged between 40 and 59 and 4 aged over 60; 1 person has a secondary school diploma and the others (N=16) have a university degree. In a comparative perspective, given the number of participants and their heterogeneity, more attention was paid to: gender (M/F) and geographic areas in terms of the three EU countries selected. The following table ( Table 3) shows the composition of the sample relating to these two characteristics that are used as descriptive variables. 

**Table 3.** Workshop participants by gender and Country.

	F	M	Total
Italy	21	13	34
Netherlands	6	7	13
Austria	12	5	17
Total	39	25	64

Considering also the self-exclusion phenomenon, the sample is not representative of the society of each country, but it can be considered as plural and inclusive as possible. It must be noted that the organisation of the workshops coincided with the second wave of the Covid-19 pandemic and the related lockdown and restrictions, which hindered the development of a more representative sample of participants for the workshops.

For each country the participants were divided into groups. For Italy, 4 groups of 8–9 people were made; for the Netherlands 3 groups of 4–5 people; finally, 4 groups of 4–5 people were created for Austria. These sub-groups were defined in advance during the selection process to balance the interaction between people with different socio-demographic characteristics.

### Ethics and consent

This project has been ethically approved via the Institutional Review Board project coordinator. The three workshops were fully recorded; each introduction, sub-group discussion and plenary conclusion was captured in audio and video with the consent of the participants. Participants gave their informed written consent for the use and publication of their data and the transcripts were not returned to participants for comment and/or correction.

### Data analysis

Each moderator provided the transcription of their workshop and the analysis of the same. In total, there were three encoders. Using the questions as guidelines for the analysis, the coding tree was provided by the authors in advance; however, the themes identified are derived from the data without the application of predetermined theoretical categories. In fact, using the constructivist approach of Grounded Theory ( Charmaz, 2006; Glaser & Strauss, 1967), the transcripts were analysed to derive new insights from the data. The process of coding suggested by this approach is often divided into three phases: *open*, *axial* and *selective* coding ( Charmaz, 1995; Pidgeon & Henwood, 1997; Strauss & Corbin, 1998). Summarising, a recursive process is implemented and in each phase of coding the data is de-constructed and collected under a label (code) in order to create some categories of meaning. These categories present in an organised way the most relevant statements of the participants.

In this way it is possible to observe the different themes that emerged from the group discussions and identify the central elements around which the participants expressed their opinion. The trustworthiness and credibility of the data analysis processes has been ensured by inter-judicial triangulation ( Denzin, 1989; Denzin & Lincoln, 2011; Flick, 2018) which is based on a constant and fruitful exchange and comparison between researchers. In fact, as will be seen in the presentation of the results, there are some elements of *convergence* and *complementarity* of the results between the different workshops, especially in relation to the geographical variable.

## Results

Following the structure of the workshop, the results were organised in four parts that recall the different phases and moments of the experience; therefore, the presentation of the results can be distinguished in the following sections: first discussion after watching the two videos without audio; second discussion after watching of the two videos with sound; news control activity; and the final dialogue phase dedicated to recommendations by the participants. The full transcripts can be found under *Underlying data*. ^13^

### First phase - video without audio and group discussion

For he first phase (video without audio), the arguments of participants can be structured in four dimensions: *topic that participants detected* within the videos without audio; *assessment of the credibility* of the videos; *emotional assessment* in which participants determined both what they were feeling through these videos as well as what possible feelings these videos evoked or tried to evoke; finally, a *critical discussion* of the two videos.

***Topic of the video.*** The first question sparked a discussion between participants about the subjects of the two videos. Several participants identified 5G and Coronavirus as the main topics of the first video, though some saw it as conspiracy theory propaganda, while others saw it as a humorous take on the subject, and on the risks of scientific communication and disinformation. Thus, participants perceived a relationship between 5G and Covid-19, but if this relationship was positive or negative still seemed to be unclear for most.

In the view of many participants the second video focused on sharing experiences from the perspective of Coronavirus patients. Similar to the negative or positive relationship between 5G and Covid-19 in the first video, participants understood the main topic, but it was less clear who were sharing these experiences and for what purposes.

Although many participants guessed the topics of the videos very closely, there was a general feeling within the groups that, in the absence of sound, there was a lack of clarity around the real subjects. In addition to image recognition and discussion, the participants tried to identify the role of the various people who appeared in the videos and this helped to frame the subject of the video.

*“About the first video which ... which intrigued me a lot, I am very curious to hear the audio, it seems on the relationship between 5G and Covid, but I do not know which might be the expressed point of view.” (Italy, Female)*
“ *The first video, which I saw, seemed to be at least about 5G and I think a link was also made there with radiation and Covid. And the second video, which I could deduce, was about Covid and the impact of Covid because they talked to people who had it.*” *(Netherlands, Female)*.

***Assessment of the credibility.*** Some comments about what does or does not make communication credible emerged in this phase. Interestingly, when assessing credibility most participants focused on the people who were present in the video (role assessment), as well as where the videos came from (source assessment). Doubt about the role of people and the source seemed to be a recurring aspect in assessing credibility as well as the style of communication.

“ *How quickly we believe things, that is often due to the name, so what credibility does a news brand have [...]. You can of course use that very well for your message, because you know that people believe you. So I think it’s interesting to also look at who produced the video*.” *(Netherlands, Male)*
“ *The second actually appears as a very calm institutional video, a very serious situation especially so that it transmits trust in who is transmitting this information.*” *(Italy, Female)*

***Emotional assessment.*** Another probe question investigated the participants’ emotional reactions to the two videos. Participants tended to agree that both videos were emotionally charged. They connected this emotional charge to the suspected topic (the pandemic) on the one hand, and to certain stylistic and visual solutions on the other hand. Feelings of manipulation, discomfort, aggression and fear were prevalent, particularly in relation to the first video. For both videos, many participants highlighted that they seemed to be manipulative; the videos were all very “over-dramatized” and this was often closely related to the idea that the videos had an “American” style. The perception of the difference between the two videos often emerged: the first was seen as much faster, more emotional and graphic, while the second was considered more calm, neutral and probably fact-based.

“ *I found the first one rather aggressive. So the presentation and the intermediate images came across as rather aggressive, whereas the second one was somehow presented in a more objective way*. *” (Austria, Male)*

***Critical discussion.*** Finally, participants expressed their critical opinion on the two videos in terms of the style, format, and imagery. The first video was largely criticised for the speed of its images, apparently unrelated between each other, and for the style this was deemed aggressive. Instead, the style of the second video was assessed as calmer and more informative, with the focus on people rather than images. Even if some images, from both videos, impressed the audience (e.g. a dog with a tinfoil hat, a speaker with a blank badge and an empty hospital bed), they still left room for interpretation, hence the need for audio. Contributing to this tug-of-war between images and the need for audio were perceptions of a lack of clarity about the purpose of the videos. The emotional impact already described also played a role in the global critical evaluation. Both videos were criticised for the manipulative feeling perceived by many participants, but the first video was rated more negatively in relation to its aggressive style.

“ *The thing that gave me a lot to think about in the first video is that you jump from one situation to another in a very unrelated way and this without hearing the audio creates confusion but it also gives me the idea that they want to create an ad hoc narration.*” *(Italy, Female)*

### Second phase - video with audio and group discussion

Five main elements emerged from the analysis of the second group discussions (video with audio): *personal assessment* in which participants assessed and critiqued their personal lenses; *new credibility assessment* of the videos; *new emotional assessment*; *new critical discussion* and review of style, format and content; *feedback and advice* for improvement of the videos.

***Personal assessment.*** Participants reflected on their personal position with regard to the topics or the images seen in the videos; they reviewed the content of the videos by relating it to their own experiences. For example, one person’s experience with Covid-19 and related thoughts about isolation had an impact on their’ trust in the speakers in the second video. Moreover, some participants noted the importance of their culture and personal frameworks. This is a very interesting finding which illustrates the personal sense-making process and that it can influence thought processes, opinions and decision making.

“ *Surely [...] with regard to trust, personal experience affects it, for example I can trust what they were saying in the second video [...] because I lived it, a month ago I got sick, I was struggling to breathe so I can say: I’ve lived it, I know what it means, I trust those people.*” *(Italy, Female)*
“ *So in that respect I think, yes, that’s a bit of a critical point. Perhaps we, as a scientific elite, are very much engaged in thinking about others.*” *(Netherlands, Male)*
“ *It’s not presented that way here. That means it has a completely different effect on us, because we are not used to this format at all, yes? And that’s why we perhaps have to take into account that we are judging something here that doesn’t fit into our classic media consumption at all.*” *(Austria, Female)*

***New credibility assessment.*** During the second session, participants once again discussed factors that support or hinder credibility. Many expressed their concerns over the credibility of the speaker in the first video, who commented on the frequent ambiguity of conspiracy theorists’ sources but did not display or introduce references himself. Regarding the second video, instead, many agreed on the fact that the direct sharing of a personal experience was credible enough. In fact, according to participants, mentioning of references in the second case could be considered less important than for the first one since the personal stories themselves serve as sources. The choice of interviewing people itself is an important element that might influence the credibility, even if it can prompt the issue about who the interviewed are or why and in which way they were selected.

Some participants stated they would not necessarily believe videos like these and that it is always hard to trust it completely. However, when the content and context was clear and the roles were more recognizable, participants stated that they perceived the videos as more credible. However, some participants tended to mention a gap between the message and the style, especially for the first video, and this discrepancy hurts the trustworthiness and credibility of the message. Credibility might be increased by providing more facts during the videos, that is, further links at the end of the first video or the opinion of experts and background facts that complement the personal tales of the survivors of the second video.

Discussing the importance of proper sources, participants added that you cannot always check the source or that a good information source was missing. Furthermore, they stated that social media as a source could possibly be more “dangerous” or “steering”. Overall, verifiable information is essential in the view of the participants; some of them underlined that one should consult many different (online or offline) sources to gain a good overview of hot and controversial topics such as Covid-19.

“ *let’s say that.. eh ... granting him [speaker of the first video] my trust is somewhat limited by the fact that there is no name, that there is not even a brief account of himself, of what skills he has.*” *(Italy, Male)*
“ *Because sometimes there are articles that look as if they are scientifically very well prepared, but when you try to find the sources behind them, sometimes you don’t find anything at all or they are simply studies that are very questionable in terms of quality. And I think that’s quite dangerous, because it looks as if it’s well researched, as if it’s a scientific article, and very few people take the trouble to research it again.*” *(Austria, Female)*

***New emotional assessment.*** Emotional assessment was found less evident than during the first discussion (video without audio), but feelings of frustration and confusion still remained. Many participants stated again how they felt the videos were very “over-dramatized” and emotional and they noted stylistic choices to build this “drama”.

“ *I still have the feeling of slight anxiety and confusion that the video sent me without audio, that’s ahem although the perception was totally different [...] the feeling of anxiety remained a little bit.*” *(Italy, Female)*
“ *What did arouse, what I eventually thought about, is that for me, those films, both 5G and the second film, cause me a kind of frustration that it is actually necessary to talk about this in such a way that people still- I don’t know how old this movie is, but these kinds of movies are still being made.*” *(Netherlands, Female)*

***New critical discussion.*** The critical assessment addresses those aspects that the participants found important (or not) and what they were still questioning in terms of style, format and content.

First, the audio was considered an important component to the videos. Participants found the videos with audio less chaotic. The audio gave them a more consistent context and it surprised a lot of the participants because of the shift in their perception of the videos’ subject, particularly with regard to the first video. Thus, the audio is able to change individual perceptions and some participants stated they were afraid to obtain wrong information as they frequently watch videos without audio on social media, highlighting the importance of this element.

In terms of the content, the participants discussed the relationship of Covid-19 and 5G once again, but this time felt they understood the direction of the relationship. Closely related to this are the discussions about the purpose of the videos. Some participants stated that the first video was trying to “prevent” people from becoming conspiracy thinkers. Instead, with the second video, participants felt there was an emphasis on the seriousness of the virus, and that this was the purpose of the videos as well as to make people aware of the relevant effects of Covid-19.

Discussing the purpose of the videos also in relation to their style, the participants highlighted that the format and style are considered very important because the chosen presentation method of a given message may highly contribute to the achievement of the purpose. In other words, style and format should also be chosen in relation to the specific purposes of the communication in order to ensure a good impact. The first video was once again perceived as more calculated rather than genuine, while the second one was felt to be calmer because it seemed like an informative report. Moreover, some participants thought the sound enhanced their emotional responses to the images and topic of the videos. For the second video, some Italian participants added that the choice of images were unnecessary to the narrative’s scope. Finally, some Austrian participants also discussed the importance of the target audience. A generational and a cultural division line was brought up: there seemed to be an agreement that the first video would be more effective for younger people and for an American audience. Older people had an aversion towards “over-dramatized” stylistic choices as presented in the first video, and in general the Austrian (and maybe Central-Eastern European) audience was not familiar with such sensationalist presentation of scientific facts. In fact, especially some Austrian participants expressed that sensationalism can be counter-productive, reducing the level of involvement and attention that the public can experience to the point of compromising the truthfulness and reliability of the information transmitted.

Particular criticisms were given in relation to the content, style and format. Some Italian participants made it very clear when they did not agree with the way the videos were framed, when the rapidity in the sequence of images and scenes was a problem or when they did not agree with the speaker’s attitude. Concerns also emerged about how issues in the videos were not properly addressed. Many participants felt a certain distress towards the content as they perceived the video to be making fun and ridiculing a certain audience, namely conspiracy thinkers. In the opinion of many participants, this way of presenting and wording the content could be a counter-productive approach since it might alienate people more receptive to conspiracy ideas.

Furthermore, the content’s presentation felt very polarising to the participants, and this was something that distracted some of them from the content itself. In relation to this, participants felt that disturbing stylistic choices were made. The first video felt loud and “screamy”, with images flashing too fast or from unusual angles. It was considered to be messy and chaotic, and participants highlighted the use of unfavourable imagery as images were restless or too slick. Finally, some Dutch participants critically questioned the video’s diversity, mainly considering the gender dimension. Participants mostly noted that men were not sharing any of the experiences or other roles were not considered. Some participants even noted how men were representing the authority roles and women were providing the emotions, which they thought the video did on purpose.

“ *At least, if I’m somewhere and I quickly check my Facebook, then I don’t have my sound on. Because then I think, those people don’t need to hear what I’m watching. But then you actually got the wrong impression. Then you have not understood the message they are trying to convey. And we actually had that with the first, with the second video [...] at first I had a different impression than the second time [...]. It gives a lot more context and you miss that without sound.*” *(Netherlands, Female)*
“ *For the second, yes it is a sad video, if we want, but it is aimed at, making information perhaps within a news program to give testimonies of people who have been through this situation, and therefore alarm citizens, in my opinion, to pay attention and behave well, so that it does not happen to them too.*” *(Italy, Male)*
“ *Yes, so for me it was also with sound, this blatant marketing, so it hasn’t really changed much for me, now from my perception. And, yes, (…) one can discuss which target group one wants to reach with it. Probably younger people, because it’s more aimed at fast editing sequences, but, yes, whether you really reach them with it, I also question that.*” *(Austria, Female)*

***Feedback and advice.*** Participants had much advice for improvement of the videos. They also had suggestions and comments about the shortcomings of scientific communication and governmental communication practices in their own country. Mostly, participants expressed the need for creating a serious discussion, and that one should be interested in and be understanding towards differing positions. Certain discussions within the Italian groups went a little off-topic and these participants felt that the problem often lies in the management of communication by the government, in particular as happened with the Covid-19 issue.

Many Austrian participants advised to improve the style and credibility of the videos, taking into account the target audiences. Even if the choice is made to have a video on Covid-19 in a more ‘infotainment’ format - as in the case of the first video - the style should not alienate the audiences interested in the pandemic and related misinformation. In contrast to the actual solutions in the first video, images and camera angles should be more relaxed and conventional, and the presenter should “tone down” his message. In regard to credibility, one cannot just discredit the conspiracy theories without providing the viewers with proper sources of information. As one participant suggested, the solution may be to mix the layman presenter with some experts refuting popular conspiracies, such as the relation between 5G and Covid-19.

Some others have referred to the fact that younger audiences should receive an “introduction to the media” to become more aware of the choices of style and format or the type of sources to avoid the risk of manipulation.

“ *Communication has changed a lot and there is always a lot of everything but above all in this moment of emergency we are overwhelmed with this flood of information [...] I mean, there is no central authority that tells us this is right, this it is wrong, there are many references and even the authorities fight continuously [...] and the situation is really ... saddening even for us who work with this because [...] we always hope that those from the upper floors may talk, that is, make a simple mental plan on how to act in these situations but in reality no [...].*” *(Italy, Female)*
“ *I’m not a psychologist, but that the people who - As soon as you contradict them, at that moment they are more likely to harden in their point of view than to actually start thinking about their point of view. The point is that you can indeed go along with them much better and say gosh, what are your arguments and why do you think this?*” *(Netherlands, Male)*
“ *Maybe you could mix it [the second video] up with experts speaking. So that, for example, you first bring up how Corona could affect the lungs, for example. And then, the lady tells how she felt. That it was difficult for her to breathe. And so on. That you first bring the facts and then the emotion. Or the other way round, first the emotion and then the facts. That it simply gets more substance.*” *(Austria, Female)*

### Third phase - Fact-checking

After the video sessions with discussion, an activity on fact-checking approaches and media platforms followed: participants were asked what their sources of information were ( *Where would you go to check and find more information about the topic?*) and then they had five minutes to look into a pre-selected fact-checking website. Fact-checking websites were different for each country because they were context-based; they are shown in Table 4.

**Table 4.** Fact-checking websites.

	Fact-checking websites
Italy	BUTAC: https://www.butac.it/
Netherlands	Nieuwscheckers https://nieuwscheckers.nl Netwerk Mediawijsheid https://www.mediawijzer.net net
Austria	APA Faktencheck: https://apa.at/faktencheck/ueberblick/ ueberblick/ Mimikama: https://www.mimikama.at

Groups discussed their approaches to fact-checking and many participants shared the best practices they had learned and gotten used to implementing, especially during recent times. Regarding sources of fact-checking, several differences between the three countries were found.

Italian participants cited institutions, news programmes and regulatory bodies for communication as generally trusted sources for news. Some reflected on individual responsibility when sharing news, illustrating how they resort to verifying the veracity of the information they are sharing (e.g. via Research Gate).

With regard to Dutch participants, many of them considered their relations’ network highly important. Talking about news items with either family or friends creates interesting discussions in their opinion. Another approach was reaching out to someone in their network with a certain profession to get more information on a topic. Using the fact-checking websites that were recommended, participants were asked to review the websites and to critically reflect on using them. The main outcomes of these discussions were that they had to be more user-friendly and easier to find. One respondent highlighted that he would like for social media platforms to do the fact-checking for them and put the sources underneath so you could immediately see if something was true or not. Another person highlighted the importance of visuals. A case in point is the Instagram stories of *Nederlandse Omroep Stichting* (NOS) ^14^, a Dutch public broadcasting news channel. These stories are fast paced, compact and highly visual. Participants considered this very successful, especially for younger audiences.

Austrian participants mentioned mostly “general” online sources, such as Wikipedia, Google Search or Google Scholar. The online versions of (German-speaking) trustworthy traditional newspapers were also mentioned. Some people said that they use social media as a news source (e.g. Facebook) by following famous people and/or friends about whom they assume that they have a better overview of certain topics. Few participants said that they watch videos, e.g. on YouTube. Interestingly, more traditional sources such as newspapers, radio or television were rarely mentioned. These participants highlighted that, since most people have neither the time nor the inclination and background knowledge to assess the credibility of such sources, the responsibility of moderators or journalists has been increasing. When a website has a good reputation already built up, it is more likely that it will be trusted by the participants. Some of these participants mentioned that they try to check more diverse sources; in this way they get a better picture of issues they are really interested in (“get out of their bubbles”) but this kind of checking may take up much time. In this regard, various online fora were mentioned that are checked by some participants even though they might not agree with the general direction (such as fora giving place for conspiracy theorists) but are simply interested in what is “out there” in the online space.

In general, a returning subject was that of media awareness, especially among young people, and how it is essential that they are educated about the framing of (social) media. Indeed, participants commented on how difficult fact-checking can be for the general public, and how rather few citizens possess the skills to successfully navigate the web and use it to fact-check all information they may come across. Furthermore, education level was considered to be important for interpreting information, as well as for discussion styles and how both the media and citizens should keep this in mind to counteract the emergence of “filter bubbles”.

### Fourth phase - Recommendations

During the final phase of the workshop, participants shared numerous comments and recommendations on how to improve science communication. Many addressed how to engage in reliable and credible science communication. Some were country specific and others more cross-cutting. The results can be organised in two main groups:

- *Source*: to communicate credibility it is important to cite the source and affiliation or background of the communicator. Communication can be more credible when it resonates with the personal experiences of the audience, however participants also recognized the relevance of remaining critical and not believing information just because it fits their world view. The communicator should critically ask themselves what their intention is and try to remain neutral by sticking to objective facts and not personal opinions. Polarisation also does not have a good effect on credibility. Audiences are sensitive to the communicator’s intentions and if a topic is presented from only one perspective it can create a feeling of manipulation. On a similar note, participants indicated the need to involve more points of view in the debates, giving space to different positions or, in addition, using different sources to obtain a more complete picture.
- *Format and style*: the timing and density of information should be appropriate for the audience because members of the public would like to have enough time to process the information and form their own opinion. In this sense, the length and speed of the video should be adequate, and the style should be engaging, taking care not to be too elitist. In general, the target group is important, and media content should be established taking into account the specific needs and characteristics of consumers. More specific indications referred to gender and contexts of origin of the audience. For example, “American” style multimedia content may not be ideal for informing and influencing European audiences as that style may not resonate with one’s cultural habits in terms of the kind of format and style of communication with which people usually interact in their everyday life.

## Conclusion

Starting from the initial consideration about the global pandemic that amplified the relevance of science communication in society ( Metcalfe *et al.,* 2020) and the increasing use of the visual through the media ( Hermann *et al.*, 2021; Kümpel, 2019; León & Bourke, 2018; Meier *et al.*, 2020; Trillò *et al.*, 2021), the workshops triggered a debate about various aspects of communication perception and investigated how the public perceives and evaluates science communication. The particular type of activity proposed with the analysis of video materials without and with audio made it possible to highlight some relevant aspects concerning the explanatory power of the audio and of the images.

Two types of videos were used and the differences in format and style, also related to the trustworthiness issue, were immediately identified by the participants. They define the first video – with a narrator that talks about the connection between Covid-19 and 5G – as chaotic and less reliable; instead, the second video - interviews with patients in a hospital setting - was considered calmer and more professional.

Video and audio are the two elements on which we focused our attention. The videos propose some stereotypes and contribute to instilling images in people’s memory and imaginary; the audio is necessary to understand what the videos are exactly about. There is a gap between video and audio. Our results are in line with the arguments of *Framing Theory* theorists ( Fahmy, 2004; Kress & Van Leeuwen, 2001; Nilsson, 2015; Parry, 2010), images could be misleading and can induce interpretations quite different from what is intended to be transmitted. In this perspective, the power of images turned out to be very strong especially if we consider some characteristic images that are also very much rooted in the imagination of the public such as the tinfoil hat that should “protect” from radiation, radio towers seen as a source of diffusion of something fearsome, or the hospital beds often proposed as a symbol of illness.

The divergence between video and audio creates a rift in credibility and trust in the information proposed. Thus, what is necessary for credibility and trust? Sources and reliability are the main elements that need to be taken into account. For example, our results underline the need to have objective information from official sources that are easy to reference. Credibility might be increased by providing more facts during the video or referring back to sources. Participants discussed the importance of proper sources and added that, while their own research and curiosity is of utmost importance, many different sources (online and offline) should be checked to gain a good overview on such topics. However, based on the fact-checking exercise, it seems that the participants themselves lack good awareness and extensive knowledge about fact-checking practices or websites (with slight differences across countries, with Austrian participants seeming to be the most aware of such platforms).

Other key takeaways from the analysis refer also to the powerful role of emotions in people’s understanding of science, and the need for sharing the responsibility of communicating science between different stakeholders.

The powerful role of emotions in science communication is common across the three countries participating. It is widely acknowledged that their presence promotes attention and facilitates absorption of content; many participants recognise that without emotions it is difficult to connect with the intended message, and without emotions they would easily switch off the video and change the source of information. On the other hand, we can underline the numerous observations and criticisms made about the overly aggressive communicator present in the first video; there is a widespread view that the same emotions can have counter-productive effects and more balanced and reassuring figures are preferred to talk about scientific issues, especially in the case of health. The well-known ambivalence and the double effect concerning the emotional aspects suggests a cautious attitude and a careful use of this element during communication.

Quite a few participants, across all groups, have expressed their awareness about their own responsibility as communicators of science. This role, as they say, implies a great deal of responsibility, and it is necessary to promote this consideration. It is an important basis on which to build a call for more citizen involvement in trustworthy, reliable and engaging science communication.

Are we facing a shift from citizen science to citizen science communication? This could lead to a shift in the mainstream research interests within citizen science towards more participatory forms of science communication, making citizens even more relevant actors in this context. In this way the communication of science would become the central topic of the research and no longer an “add-on” in the discussion. These kinds of considerations are relevant, especially, when the different contexts and cultural backgrounds of the citizens involved are considered. Among these differences, we underline the importance of the gender perspective that emerged during the Dutch workshop. In fact, it should not be overlooked that very often in the public communication of science, there is a tendency to perpetuate some static models such as the traditional family model where the male is the authoritarian and the female is the nurturer. Citizens, through their communication of science, can contribute to fostering these kinds of models and, in light of the possibility of a transition to citizen science communication, we should pay more attention to this risk as well. In other words, citizens are active subjects of communication with powerful means and they can be multipliers of news, even false news, through social networks. This awareness emerged strongly during the workshops and should always be kept in mind when thinking about scientific communication.

So, what kind of science is being communicated? What image of science do we have in mind when we turn our interest to science communication? ( Besley & Nisbet, 2013). And what can we conclude about the role of audio and video connected to trust in scientific communication?

Digital and visual communication have changed in recent years along with the way citizens view news and scientific communication. Images are increasingly a central element for transmitting information and citizens perceive them as vehicles of meanings that contribute to confirming uncertain visions of science. This collection of images is not enough to activate effective trust mechanisms. Although innovations in technology and storytelling brought new approaches in science and knowledge communication, text and audio are interdependent and together they remain a constitutive element of this kind of communication. In other words, the audio of visual communication is still necessary for a complete understanding of the scientific issues.

Visual communication is strongly interlinked with digital tools and the way people use it in their everyday life. From this perspective we should overcome a conception of the citizen as a “typical user” who consumes scientific news uncritically. Rather, the theoretical framework should be open to empirical measures of values such as active citizenship or resilience based on observable data. Citizens should not remain users, but should have active communication roles if we want to move towards a more relevant communication of citizen science with active citizens, transmitting and disseminating values, norms and examples.

In conclusion, we can argue that the present study has no presumption of generalisation of the results due to the nature of the research conducted and the specific number and composition of the sample. However, this is not to be considered a limitation as our results are added to a line of research within which knowledge proceeds on the path of the quality of the data collected and of the analysis processes that broaden the point of view on the issue under consideration. Further studies could go in the direction of deepening the interaction between audio and video by creating different combinations of audio-video sequences and investigating how the two communication channels can be connected if presented in different succession.

## Data Availability

DANS-EASY: TRESCA - WORKSHOPS_TRANSCRIPTS. https://doi.org/10.17026/dans-xrv-m9kn. ( Piccolo, 2022) This project contains the following underlying data: Transcripts Austrian WS Transcripts Dutch WS Transcripts Italian WS (Transcripts of the TRESCA project workshops, held in Italy, the Netherlands and Austria This project contains the following extended data: Interview guide and Field notes Evaluation questionnaire Informed consent form Consolidated criteria for reporting qualitative studies (COREQ): 32-item checklist Standards for Reporting Qualitative Research (SRQR) Data are available under the terms of the Creative Commons Zero “No rights reserved” data waiver (CC0 1.0 Public domain dedication).

## Footnotes

^2^ Consejo Superior de Investigationes Cientificas, Institute for Policies and Public Goods Dept. of Science and Innovation - Madrid (Spain).

^3^ *No, 5G Is Not Causing Coronavirus* ( https://www.youtube.com/watch?v=KassIV7qLGk)

^4^ *Do not ignore this: COVID-19 survivors speak out during DeLand round table* ( https://www.youtube.com/watch?v=FlH5DKNsuy0)

^5^ The moderators were: (removed for anonymity); they were two females and 1 male. They have a PhD, work as researchers and have a great deal of experience in group facilitation; in particular, for these research they had a specific training with (removed for anonymity) who designed the workshop.

^6^ Piccolo, dr C. (Observa - Science in Society) (2022): *TRESCA - workshops_transcripts*. DANS. https://doi.org/10.17026/dans-xrv-m9kn

^7^ Piccolo, dr C. (Observa - Science in Society) (2022): *TRESCA - workshops_transcripts*. DANS. https://doi.org/10.17026/dans-xrv-m9kn

^8^ Observa - science in society: https://www.observa.it/

^9^ Erasmus University Rotterdam: https://www.eur.nl/en

^10^ Zentrum für Soziale Innovation (Centre for Social Innovation): https://www.zsi.at/

^11^ Participants exercised the rights defined by Regulation 2016/679/UE GDPR ( https://eur-lex.europa.eu/eli/reg/2016/679/oj). According to data protection legislation, organizers informed participants that the legal basis applied in order to process their personal data was found in the Organisation’s Privacy Notice ( https://www.eur.nl/en/disclaimer/privacy-statement). Participants signed a consent form and they were informed that the data would be processed in a cumulative and anonymous form for research purposes only.

^12^ Gender; Age; Level of individual finalized studies; Geographic areas; Rural or Urban backgrounds[Following the EU regulation, we consider urban areas a minimum population of 5.000 inhabitants]; Cultural minorities representation [Following the Council of Europe Recommendation 1201 (1993)].

^13^ Piccolo, dr C. (Observa - Science in Society) (2022): *TRESCA - workshops_transcripts*. DANS. https://doi.org/10.17026/dans-xrv-m9kn

^14^ Dutch Broadcasting Foundation.
