# Supplementary material for: Experimenting with visual content: online focus group on citizens’ perception and trust in science communication
Source: Open Res Eur. 2023 Mar 30;2:60. Originally published 2022 May 18. [Version 2] doi: 10.12688/openreseurope.14621.2 (PMC10445909; doi:10.12688/openreseurope.14621.2)
Supplement: Peer review history

# Peer review: Reviewer response for version 2

**DOI:** 10.21956/openreseurope.17066.r31088

**Published:** 2023-04-21

**Authors:** Carolina Llorente Lope

**Affiliations:** 1 Studies Centre on Science, Communication and Society, Department of Experimental and Health Sciences, Universitat Pompeu Fabra, Barcelona, Spain

I have no further comments to make

If applicable, is the statistical analysis and its interpretation appropriate?

Yes

Is the study design appropriate and is the work technically sound?

Partly

Is the work clearly and accurately presented and does it engage with the current literature?

Yes

Are the conclusions drawn adequately supported by the results?

Yes

Are sufficient details of methods and analysis provided to allow replication by others?

Partly

Are all the source data and materials underlying the results available?

Yes

Reviewer Expertise:

Science communication, public engagement, responsible research and innovation

I confirm that I have read this submission and believe that I have an appropriate level of expertise to confirm that it is of an acceptable scientific standard.

# Peer review: Reviewer response for version 1

**DOI:** 10.21956/openreseurope.15787.r30543

**Published:** 2023-01-26

**Authors:** Letizia Caporusso

**Affiliations:** 1 Department of Economics and Management, Università degli Studi di Trento, Trento, Italy

The paper discusses the relative importance of visual and verbal stimuli – and their implicit (in)coherence – on people’s perception of science communication credibility and reliability. The comments below should be interpreted as (hopefully useful) insights for forthcoming projects. I appreciate this contribution as a preliminary study, and as such its contents are worth indexing: I should leave to the authors to decide whether they want to enrich the present version with some amendments and integrations on the issues that I raised.

**Introduction**. Authors pose that visual stimuli have become paramount in contemporary society and the combination of formats, styles, choice of images together build up a shared visual imaginary. According to framing theory such imaginary can purposedly be manipulated.

 Perhaps in this section the authors might want to consider also the individuals’ agency and their ability to choose, combine, compare, integrate different (private and institutional) sources. Some passages (here and in the following paragraphs) seem to echo the hypodermic theory.

 I would also be careful in referring to the “experimental character” of this research, as the design does not contemplate any kind of control group.

**Methods**. This section is thoroughly described and the main concern is about the lack of a “reverse design”: all groups were provided with the video-only stimulus followed by the video plus verbal stimulus. Further studies might want to test what happens if a verbal-only stimulus is followed by a verbal plus video stimulus and compare the ensuing discussions. Some kind of interaction between the audio and the video is also very likely: it might be interesting to test a message which is coherent with the interviewees expectations, against an incoherent one (perhaps with some reference to dissonance theory and its effects).

 The paper indulges in length in the method description, but the participants profile is described only according to their gender. More information about their level of education and age group might give more insight and grounding to the findings,

**Data analysis.** To what extent did the participants ideas converge or diverge within the groups? Why? Differences amongst countries are being taken into consideration but little is said about different positions that might (or might not) have emerged during the discussions, and whether they were related to any relevant socio-anagraphic characteristic. 

**Results.** This is a descriptive and narrative paragraph. In future works the authors might want to consider how the elicited dimensions interrelate with each other. Perhaps more consideration might be devoted to addressing the issue of stereotypes and prejudice (how do people portray what is scientific, credible and trustworthy?)

**Conclusions**. In this section the authors interpret the results above described and connect them to the theoretical background anticipated in the introductory section. I am just not totally confident in the conclusion according to which participants were mostly unaware about fact-checking practices: how does this emerge from the verbalization? Was this conclusion referred to the specific knowledge of the proposed fact-checking websites or is this a more far-fetching inference?

*Minor typos:* correct a repetition (“evaluation evaluation”) in the tools and procedures section and add a closed parenthesis in the last line of the section “underlying data"

If applicable, is the statistical analysis and its interpretation appropriate?

Not applicable

Is the study design appropriate and is the work technically sound?

Partly

Is the work clearly and accurately presented and does it engage with the current literature?

Yes

Are the conclusions drawn adequately supported by the results?

Partly

Are sufficient details of methods and analysis provided to allow replication by others?

Yes

Are all the source data and materials underlying the results available?

Yes

Reviewer Expertise:

methods and techniques of social research; sociology; citizens participation and engagement; market analysis and marketing

I confirm that I have read this submission and believe that I have an appropriate level of expertise to confirm that it is of an acceptable scientific standard.

# Response

**Published:** 2023-03-20

**Authors:** Chiara Piccolo

**Affiliations:** Observa - Science in Society, Italy

Reviewer 2: The paper discusses the relative importance of visual and verbal stimuli – and their implicit (in)coherence – on people’s perception of science communication credibility and reliability. The comments below should be interpreted as (hopefully useful) insights for forthcoming projects. I appreciate this contribution as a preliminary study, and as such its contents are worth indexing: I should leave to the authors to decide whether they want to enrich the present version with some amendments and integrations on the issues that I raised.

**Introduction**. Authors pose that visual stimuli have become paramount in contemporary society and the combination of formats, styles, choice of images together build up a shared visual imaginary. According to framing theory such imaginary can purposedly be manipulated.

 Perhaps in this section the authors might want to consider also the individuals’ agency and their ability to choose, combine, compare, integrate different (private and institutional) sources. Some passages (here and in the following paragraphs) seem to echo the hypodermic theory.

 I would also be careful in referring to the “experimental character” of this research, as the design does not contemplate any kind of control group.

**Answer: Studies conducted by Scheufele and Iyengar permit to verify to what extent the media can influence public opinion (Scheufele & Iyengar, 2017). For example, new digital technologies (e.g., morphing techniques) can be applied to measure precisely which manipulation leads to specific effects. In this way, some algorithms related to the use of big data and in particular to Facebook, facilitate the study of which visual messages allow the public to act autonomously while maintaining a critical look.** **The proposed activity was designed as a quasi-experiment. Unlike a real experiment, a quasi-experiment is characterized by a series of characteristics, as indicated by Campbell & Stanley (1963): manipulation of the variables examined, the absence of a control group, the non-randomness of the selection of the subjects involved and the formulation of hypotheses that indicate a relational link between associative variables (covariation).**

**Methods**. This section is thoroughly described and the main concern is about the lack of a “reverse design”: all groups were provided with the video-only stimulus followed by the video plus verbal stimulus. Further studies might want to test what happens if a verbal-only stimulus is followed by a verbal plus video stimulus and compare the ensuing discussions. Some kind of interaction between the audio and the video is also very likely: it might be interesting to test a message which is coherent with the interviewees expectations, against an incoherent one (perhaps with some reference to dissonance theory and its effects).

 The paper indulges in length in the method description, but the participants profile is described only according to their gender. More information about their level of education and age group might give more insight and grounding to the findings.

**Answer: The suggestion of a "reverse design" is very interesting and could be considered in further studies. The practical conditions in which the project was carried out (e.g. times, deadlines; costs) required some methodological choices to be made; in agreement with the literature, we decided to use this design: the video-only stimulus followed by the video plus the verbal stimulus.** **The suggestion of testing a message consistent with respondents' expectations against an inconsistent one, using dissonance theory, could also be interesting. It is evident that in this case the design of the study should be very different because it should foresee a preliminary study on what could be consistent and what not for the selected participants. Also in this case, the method choices went in another direction considered more in line with the project as a whole.** **Some of these considerations have been added in the conclusions section as possible further studies.** **We added a more detailed description of the participants regarding their socio-demographic characteristics. In any case, we have to highlight that, given the number of participants and their heterogeneity no statistically significant correlations were investigated. Variables “gender” and “country” were considered only as a descriptive variable.**

**Data analysis.** To what extent did the participants’ ideas converge or diverge within the groups? Why? Differences amongst countries are being taken into consideration but little is said about different positions that might (or might not) have emerged during the discussions, and whether they were related to any relevant socio-anagraphic characteristic. 

**Answer: This research ranks among the qualitative studies and having a "representative sample" was not our aim. The socio-demographic characteristics that we have collected are useful for better framing the results and, given the typology of the sample, we have chosen to focus on two main descriptive variables: "gender" and "country". For these reasons we have not taken into account the different positions linked to any other socio-personal characteristics. Nothing limits the possibility of doing it in another studio. How participants' ideas converge or diverge within groups is shown in the "Results" section.**

**Results**. This is a descriptive and narrative paragraph. In future works the authors might want to consider how the elicited dimensions interrelate with each other. Perhaps more consideration might be devoted to addressing the issue of stereotypes and prejudice (how do people portray what is scientific, credible and trustworthy?)

**Answer: It is an interesting suggestion that we can take into consideration for the next studies.**

**Conclusions**. In this section the authors interpret the results above described and connect them to the theoretical background anticipated in the introductory section. I am just not totally confident in the conclusion according to which participants were mostly unaware about fact-checking practices: how does this emerge from the verbalization? Was this conclusion referred to the specific knowledge of the proposed fact-checking websites or is this a more far-fetching inference?

**Answer: Yes, it emerged from "Step Three - Fact Checking" as detailed in that section. We recognise that we have made a generalization in the conclusion that is poorly supported by our data and for this reason we have modified the text: now the statement is connected with our participants and not with the "general public".**

**Minor typos**: correct a repetition (“evaluation evaluation”) in the tools and procedures section and add a closed parenthesis in the last line of the section “underlying data" **Answer: Done.**

# Peer review: Reviewer response for version 1

**DOI:** 10.21956/openreseurope.15787.r29320

**Published:** 2022-06-14

**Authors:** Carolina Llorente Lope

**Affiliations:** 1 Studies Centre on Science, Communication and Society, Department of Experimental and Health Sciences, Universitat Pompeu Fabra, Barcelona, Spain

The article is relevant to the community. It presents interesting data about an experiment in audiovisual scientific communication.  As it stands however, the manuscript has three gaps in my view:

**Introduction: **I think that it would be beneficial for the readers to add more contextual information in order to better understand the elements that appear in the conclusion/discussion. 

**Methods:** A more detailed description of the workshop participants would be needed. What profile did these participants have? Neither is it specified what profile of people make up the waiting list, nor if there was homogeneity between the participants in the different workshops. The authors mention that they use "several methods" for the recruitment of participants, but they only specify the "snowball technique", it would be useful to have more information about the other methods used. I would also like to know if the gender or age variables have been taken into account both for the selection process and for the data analysis.

**Results: **I would like to know how many people confirm the elements that are described in the results section. It is a qualitative study, and all the contributions are important, but for the reader it is interesting to know if a contribution is made exclusively by one person or if it is supported by more. It would also be interesting to highlight similar aspects or differences between the three countries (if any).

If applicable, is the statistical analysis and its interpretation appropriate?

Yes

Is the study design appropriate and is the work technically sound?

Partly

Is the work clearly and accurately presented and does it engage with the current literature?

Yes

Are the conclusions drawn adequately supported by the results?

Yes

Are sufficient details of methods and analysis provided to allow replication by others?

Partly

Are all the source data and materials underlying the results available?

Yes

Reviewer Expertise:

Science communication, public engagement, responsible research and innovation

I confirm that I have read this submission and believe that I have an appropriate level of expertise to confirm that it is of an acceptable scientific standard, however I have significant reservations, as outlined above.

# Response

**Published:** 2023-03-20

**Authors:** Chiara Piccolo

**Affiliations:** Observa - Science in Society, Italy

Reviewer1 The article is relevant to the community. It presents interesting data about an experiment in audiovisual scientific communication.  As it stands however, the manuscript has three gaps in my view:

**Introduction**: I think that it would be beneficial for the readers to add more contextual information in order to better understand the elements that appear in the conclusion/discussion. 

**Answer:** **We added some information regarding the theoretical framework: Scheufele & Iyengar (2017) and Campbell & Stanley (1963).**

**Methods**: A more detailed description of the workshop participants would be needed. What profile did these participants have? Neither is it specified what profile of people make up the waiting list, nor if there was homogeneity between the participants in the different workshops. The authors mention that they use "several methods" for the recruitment of participants, but they only specify the "snowball technique", it would be useful to have more information about the other methods used. I would also like to know if the gender or age variables have been taken into account both for the selection process and for the data analysis.

**Answer: We have used two methods: the snowball technique and targeted emails. We modified the text in order to clarify that there are no “several methods” but these two. We added a more detailed description of the participants regarding their socio-demographic characteristics. In any case, we have to highlight that,  given the number of participants and their heterogeneity no statistically significant correlations were investigated. Variables “gender” and “country” were considered only as a descriptive variable.**

**Results**: I would like to know how many people confirm the elements that are described in the results section. It is a qualitative study, and all the contributions are important, but for the reader it is interesting to know if a contribution is made exclusively by one person or if it is supported by more. It would also be interesting to highlight similar aspects or differences between the three countries (if any).

**Answer: We have tried to balance the legibility of the text with the density of the information. In the results section, the voices of the different participants are reported in the most harmonious way possible, highlighting when a position was supported by a minority, by participants who all came from the same country or if it was more widely shared. There was no discussion of the results with the participants. However, as written in the method section, the data analysis was a shared process among the researchers, many of whom were the same ones who conducted the workshops.**
